# Non-Coding RNA: Architects of Cellular Complexity and Agents of Malignancy

**DOI:** 10.3390/genes17030304

**Published:** 2026-03-02

**Authors:** Amil Shah

**Affiliations:** Department of Medicine, University of British Columbia, Vancouver, BC V5Z 1M9, Canada; amil.shah@ubc.ca

**Keywords:** tumorigenesis, non-coding RNA, genome regulatory network, attractor state, systems biology, cancer gene

## Abstract

Non-coding RNAs (ncRNAs) are conserved in the genome of cells across the three domains of life. They comprise a diverse group that are particularly prominent in metazoans where they provide a crucial interface between genes and proteins, participating in key cellular processes at different levels: from control of DNA transcription to modulation of messenger RNA stability to modification of protein activity. The interactions of ncRNAs with one another as well as with other RNAs, DNA and proteins form the basis of a genome-wide regulatory network (GRN). Because of the mutual influence of its components on each other, the GRN is a dynamic system. Further, the GRN imposes constraints on which genes are expressed and when, leading to specific gene-expression patterns or transcriptomes. The configurations of the activities of all gene loci represent self-stabilizing cell states, referred to as “attractor” states, each of which corresponds to a distinct cell type. The cancer cell is also an attractor state that arises from a change in the topography of the epigenetic landscape caused by dysregulation of the GRN. It is proposed that the transition to a neoplastic attractor state is caused by ncRNA alterations, while subsequent somatic mutations of oncogenes and tumor suppressor genes drive cell proliferation and clonal expansion.

## 1. Introduction

According to the prevailing somatic mutation theory, a cancer develops in a step-wise manner through the successive accumulation of mutations in a single cell, whose descendants are selected for the progressive traits of malignancy: uncontrolled cell proliferation, clonal expansion, invasion of adjacent tissues, and eventual spread to distant organs (metastasis). This model, which mechanistically links cancer pathogenesis to oncogenic mutations via linear molecular pathways, has recently been questioned [1,2,3]. Although it is generally suggested that just a few (four or five) mutations drive tumorigenesis, there is an enormous diversity of genetic alterations in cancers to the point where almost every gene has been linked to cancer [4,5]. Also, it is not usually clear which of these mutations are the main cancer drivers, since similar cancers in different individuals display only modest overlap in their gene mutations, and they may not even share any mutation in the same gene [6,7,8,9,10]. Moreover, it is not always possible to point to the specific malignant trait that an individual cancer gene induces. Although it was anticipated that a common set of driver mutations would be identified for each cancer type, this did not turn out to be the case [11]. Even more telling is the association of well-known cancer genes with common benign conditions, such as nevi, seborrheic keratosis and rheumatoid arthritis [12,13,14,15,16]. Along the same lines, somatic *FGFR3* mutations are drivers of some malignancies, such as bladder cancer and multiple myeloma; yet, curiously, germline inheritance of *FGFR3* alterations causes dwarfism syndromes, but does not increase cancer risk in affected individuals [16,17,18]. A further unexpected finding is that somatic mutations are prevalent in healthy tissues of ageing persons, who do not have a diagnosis of cancer [19,20,21,22,23,24,25,26,27].

These observations call into question the precise role of oncogenes and tumor suppressor genes in cancers, benign diseases, and, for that matter, normal ageing. Although mutations of these genes are involved in tumorigenesis, their role appears to depend on additional events or existing cellular conditions [2]. This article proposes that cells in multicellular organisms are organized by an elaborate, dynamic genome-wide regulatory network (GRN), in which non-coding RNAs (ncRNAs) play a crucial role, and whose disruption causes the cell to transform into a neoplastic phenotype. It should be mentioned that a detailed account of the structure, function and biogenesis of the ncRNAs is beyond the scope of this review, but the reader is directed to recent publications on the subject by Leitão [28], Mattick [29], Chen [30], Statello [31] and Kim [32].

## 2. Non-Coding RNAs Across the Domains of Life

A significant discovery over the past few decades is that ncRNAs, which were initially thought to be by-products of RNA processing or represent transcriptional noise, are, in fact, essential for vital cellular processes [33]. They are ubiquitous in all three domains of life: domain Bacteria (kingdom: Eubacteria), domain Archaea (kingdom: Archaebacteria), and domain Eukarya (kingdoms: Protista, Fungi, Plantae and Animalia). Across all of these, they regulate gene expression as well as buffer or enforce gene activities.

In bacteria, ncRNAs, called small RNA regulators (sRNA), are typically 50 to 400 nucleotides (nt) in length. Individual sRNAs affect the expression of a single or multiple genes that encode enzymes, transcription factors and virulence factors, establishing a hierarchical order of regulation in various metabolic and physiological processes [34]. This occurs by different mechanisms, such as altering RNA conformation, base pairing with other RNAs, or binding to proteins and DNA [35]. In general, sRNAs fall into two classes: *cis*-encoded antisense sRNA, which overlaps a segment on the opposite strand of a gene and shares an extended region of complementarity with its target messenger RNA (mRNA); and *trans*-encoded sRNA, which is encoded at a different genomic location from its target mRNA and has less complementarity with it [36,37]. Limited complementary allows *trans*-encoded sRNAs to base-pair with multiple genes. Their binding at the 5′ end or 3′ end of their target mRNAs modifies their secondary structure, exposing the ribosome binding site (RBS), thus enabling translation. Conversely, the interaction between sRNAs and target mRNAs can inhibit translation; this is brought about by degradation of mRNA by endoribonuclease RNaseE, or through direct binding of sRNA to RBS [38]. sRNA can also interact with regulatory proteins, leading to conformational adaptation of the protein side-chains and RNA; this permits recognition of different RNA sequences by the same protein. An example of this type of sRNA is Csr/Rsm (carbon storage regulator/regulator of secondary metabolism), which sequesters the global translation repressor protein CsrA/RsmE from the RBS of a subset of mRNAs [39].

Archaebacteria comprise various species that survive in extreme conditions, such as hot springs and salt lakes, although they are also present in soil, oceans, marshlands and human microbiota [40,41]. They contain sRNAs that enhance their survival in hostile habitats [42]. Multiple ribosomal RNA (rRNA) copies in mesophilic archaea permit rapid adaptation to changing temperatures, while small nucleolar RNA (snoRNA)-like C/D box types in thermophilic archaea facilitate adjustment to hot environments through RNA stabilization and transfer RNA (tRNA) methylation [43]. Further, sRNAs in some Archaebacteria, in particular the *Methanosarcina* species, appear to facilitate the formation of multicellular complexes during different growth phases and in response to environmental conditions [44]. In this context, the sRNAs fine-tune gene expression in response to external stress [45].

In eukaryotes, ncRNAs are widely expressed and are broadly divided into two groups: house-keeping ncRNAs (e.g., rRNA or tRNA) and regulatory ncRNAs. The regulatory ncRNAs are, in turn, subdivided into three types: small ncRNAs (e.g., microRNA or miRNA), which are <200 nt in length; long ncRNAs (lncRNAs), which are >200 nt; and circular RNAs (circRNAs), which range in size from 100 nt to 4000 nt [29,30,31,32]. The regulatory ncRNAs control key biological processes, such as stem cell differentiation and cell fate determination (cell differentiation). They occupy specific subcellular locations where they exert their action. In the nucleus, they coordinate expression of protein-coding genes, whereas in the cytoplasm, they control protein synthesis and can tailor protein function. The ncRNA repertoire shows a significant expansion in eukaryotes, becoming most prominent in the cells of metazoans, which require additional regulators for proper development. Consistent with this notion, miRNA surges coincide with major developmental innovations: from bilaterians to vertebrates to mammals [46]. In the next section, the evolutionary leap from simple prokaryotes to complex eukaryotes is described.

## 3. From Prokaryotes to Eukaryotes: The Hidden Layer of Non-Coding RNAs

There is a good correlation between genome size and genetic capability in domain Bacteria and domain Archaea, both of which comprise prokaryotic cells. Their genomes are haploid and consist of closely packed protein-coding genes of varying sizes; for example, from 182 genes in *Carsonella ruddii* to 8602 in *Burkholderia xenovorans* [47,48]. Prokaryotes contain relatively small amounts of non-coding sequences, about 12%, which encode a small number of regulatory RNAs or function as regulatory elements that control gene expression at the transcriptional and translational levels [45,49]. In contrast, the eukaryotic genomes carry a large amount of non-coding sequences, generally ranging from 25% to 50% in simple eukaryotes to >50% in more complex fungi, plants and animals [50].

The non-coding sequences reside in the intronic and intergenic regions of DNA. Because genes were considered to be synonymous with protein coding, the non-coding sequences were initially dismissed as “junk”. This also reflected the belief that evolutionary progress required innovations in proteins that subserve cell signaling and developmental pathways. The later realization that there is, in fact, no correlation between the perceived complexity of eukaryotic organisms and the number of their protein-coding genes (referred to as the G-value paradox) questioned this assumption [51]. A further dilemma is that as cells become more complicated, a greater degree of gene regulation is necessary. In prokaryotes, the largely protein-based regulatory apparatus displays a quadratic increase in the number of regulators with genome size [52,53]. Such a system cannot be efficiently scaled because its capacity is quickly saturated. It is, therefore, inadequate for multicellular organisms where an enormous amount of information is required for the coordination of developmental pathways. It is now evident that the genomes of complex organisms produce large numbers of ncRNAs, many of which have regulatory functions [54]. In fact, the majority of the mammalian genome is transcribed in complicated patterns of interlaced and overlapping transcripts; in humans, ncRNAs account for almost 60% of the transcriptome [55].

The vast hidden layer of ncRNAs in eukaryotes answered the question about where in the genome the information that underpins biological complexity lies. The ncRNA system is aptly equipped to get around the limitations of a protein-based one because of its structural diversity and functional versatility [2,28,29,30,31,32]. That regulatory ncRNAs scale consistently with increasing cellular complexity is evidence that the evolutionary leap from simple prokaryotes to complex eukaryotes is transacted at this level [46,56]. This trend continued into the genomes of multicellular organisms, wherein the complicatedness of the ncRNA apparatus runs in parallel with developmental complexity. Consistent with this, the most evolutionarily conserved miRNAs in bilaterian organisms play a role early in embryonic development, while those that evolved later, specifically in mammals, function at later stages of embryonic development [57]. Further, species-specific miRNAs are generally active in differentiated cell types rather that during embryonic development. This supports the notion that certain ncRNAs in more complex metazoans coordinate pivotal cell activities, such as developmental timing and cell identity.

The multilayered interactions and crosstalk among the ncRNAs as well as their association with various molecular regulators, such as chromatin remodeling factors, transcription factors and nuclear trafficking modulators, create a network for relaying information and provide the groundwork for the GRN [58,59]. Some of the principles that govern the behavior of regulatory networks are examined in the next section.

## 4. The Cell from a Systems Biology Perspective

**Figure 1:**
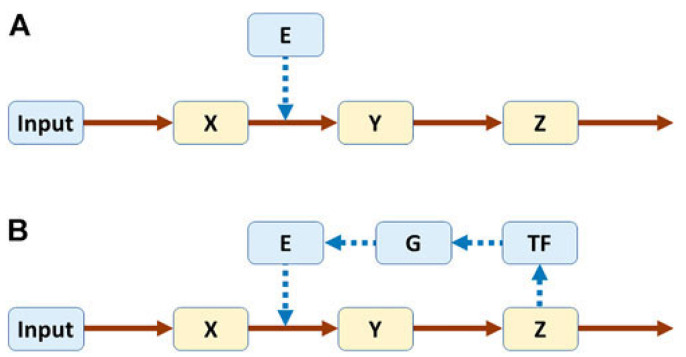
Pathways without (**A**) and with (**B**) feedback. Both systems are modeled in power-law format. Equations for pathway A: Ẋ = Input − E·X^0.5^; Ẏ = E·X^0.5^ − Y^0.5^; Ż = Y^0.5^ − Z^0.5^. For pathway B, the following equations are added: TḞ = Z^p^ − TF^0.5^; Ġ = TF^0.5^ − G^0.5^; Ė = G^0.5^ − E^0.5^. (For further details, refer to Reference [60].)

Biological systems are complex because they are made up of many components that are interactive, adaptable and dynamic. An understanding of how they function can be gleaned by dissecting specific pathways and reconstructing the whole from their individual parts. Voit described the behavior of a pathway without and with feedback loops that are central to its regulation [60]. In Figure 1, X, Y and Z represent metabolites, and E an enzyme that catalyzes the conversion of X to Y. Pathway A is not regulated, but in Pathway B, regulation is introduced with Z turning on production of a transcription factor (TF) that, in turn, causes gene G to produce E.

**Figure 2:**
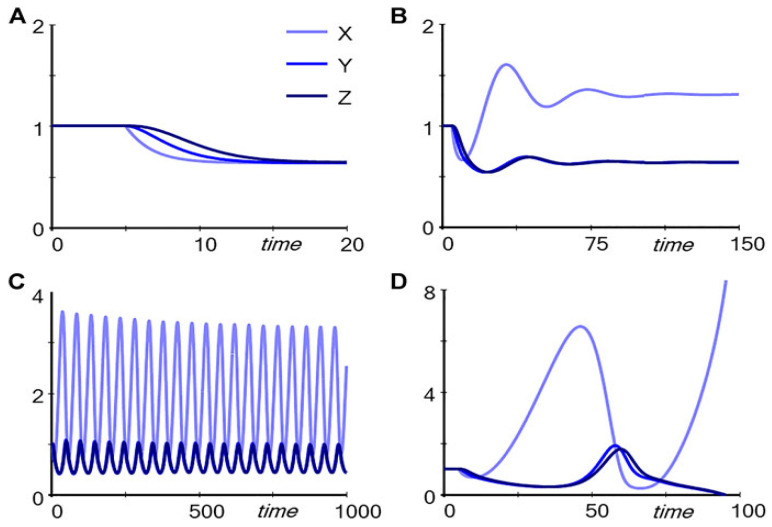
Simulation results for the two pathways in Figure 1. The system is started at the steady state (all variables at 1). At time 5, Input is reduced from 1 to 0.8. Panel (**A**): Trajectories corresponding to Pathway A (unregulated) in Figure 1. Panels (**B**–**D**): Trajectories corresponding to Pathway B (regulated) in Figure 1, with *p* = 0.4, *p* = 0.56, and *p* = 0.6, respectively. Credit: [60].

If Input is decreased, the two pathways respond differently, as depicted in Figure 2. For the unregulated pathway (Pathway A in Figure 1), reducing Input from 1.0 to 0.8, for example, causes a successive decrease in the metabolites (X, Y and Z) (Figure 2A). However, for the regulated pathway (Pathway B in Figure 1), the response follows markedly different patterns, depending on the strength with which Z drives production of TF, captured in the parameter *p* (Figure 2B–D). If *p* = 0.4, the response is dampened and the levels of Y and Z decrease (Figure 2B); for *p* = 0.56, the system settles into stable limit-cycle oscillations (Figure 2C); and for *p* = 0.6, Y and Z disappear (Figure 2D). It is noteworthy that subtle numerical alterations in the parameter setting can lead to unpredictable qualitative changes in response of the system.

**Figure 3:**
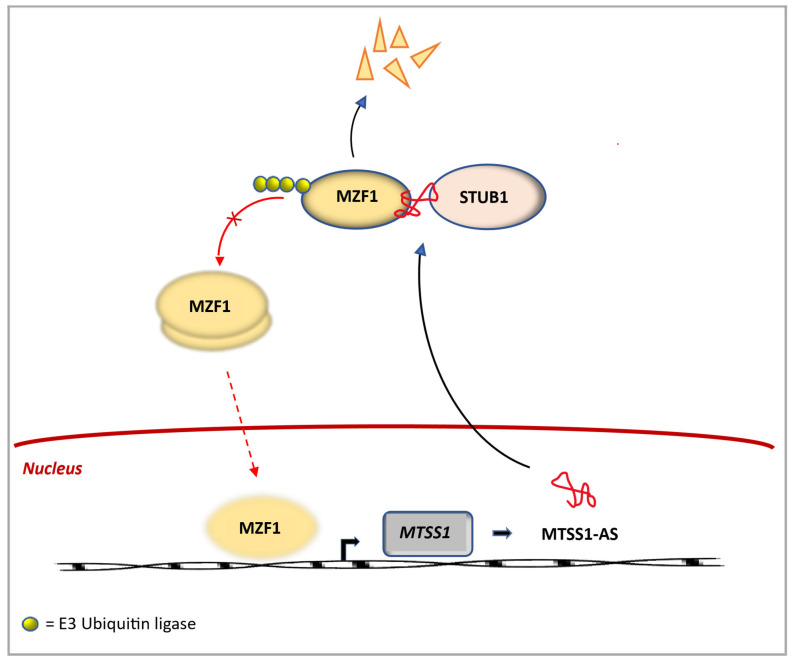
Feedback loop involving lncRNA and ubiquitin ligase. lncRNA MTSS1-AS, encoded by metastasis suppressor-1 gene (*MTSS1*), acts as a scaffold between E3 ligase (STUB1) and transcription regulator myeloid zinc finger 1 (MZFI), leading to ubiquitination-mediated degradation of the regulatory protein. Because MZF1 inhibits *MTSS1* by binding its promoter, expression of the *MTSS1* gene is upregulated.

Feedback loops that connect output signals back to their inputs are common regulatory elements in biological signaling systems [61,62]. An example is the feedback loop involved in the regulation of the metastasis suppressor-1 gene (*MTSS1*), whose encoded protein (MTSS1) is associated with cancer progression and metastasis in a variety of cancers, likely through interaction with the cell’s actin cytoskeleton [63]. In cancer, the antisense transcript, lncRNA MTSS1-AS, binds to the transcription factor myeloid zinc finger 1 (MZF1) through its nucleotide spanning positions 700–1018 (Figure 3). This enhances the interaction between MZF1 and the E3 ubiquitin-protein ligase STIP1 homology and U-box containing protein 1 (STUB1), resulting in the polyubiquitination and subsequent proteasomal degradation of MZF1. Because MZF1 inhibits the expression of *MTSS1* by binding to the *MTSS1* promoter, the decrease in MZF1 leads to upregulation of *MTTS1* expression.

Feedback loops can be positive or negative or a mixture of both (hybrid) [64]. Positive feedback causes rapid, all-or-nothing transitions, while negative feedback acts as a restorative force to create stable limit cycles, usually with faster, smaller-amplitude oscillations. Hybrid feedback promotes a broader range of oscillatory signal outputs, and with non-linear coupling of the two feedback types, novel dynamic states arise. Therefore, feedback loops are critical, allowing diversification of a system state into multiple distinct alternative states [65,66].

In classical biology, the metabolic reactions of the cell’s myriad molecules are usually reduced to linear pathways to help decipher their basic operation. Among these in mammalian species are hundreds of cell-specific signaling systems assembled from linear pathways with upstream regulators and downstream targets [67]. They are not free-standing entities, but are linked by signals within and between the different pathways. This establishes a web of connectivity that is essential in mediating important biological functions. One such function is how order is achieved during the development of multicellular organisms where determination of cell identity must be versatile, yet precise and robust to avoid errors [68]. Recently, the importance of the continuous, dynamic interactions of the cell’s biomolecules as a fundamental organizing process built into all life forms has become evident. In addition to proteins, which have long been known for their regulatory functions, ncRNAs have emerged as key components of the cell’s genome-wide regulatory system.

## 5. How Order Is Achieved: Regulatory Networks and the Origin of Attractor States

A central issue in metazoan development is how different cell types arise from the same genome of its zygote. A cell is shaped by its proteins, which perform a vast array of functions that define its traits. In turn, the collection of all active proteins in a cell is determined by its gene-expression profile at any given time. In humans, this profile encompasses the state of approximately 20,000 genes across the genome: active/inactive [69]. Since the state of every gene is governed by the GRN, it follows that the GRN oversees a vast number of theoretically possible combinatorial states, which can be conceived as an abstract “state space” [70,71]. From any theoretical starting point, the system evolves and eventually settles down into a steady state [72]. It should be noted that not all gene configurations are necessarily feasible because certain interactions may not be permitted by the regulatory constraints imposed by the GRN [73]. For instance, genes that mutually repress each other’s expression cannot be fully co-expressed (as illustrated in the example of *MTSS1* regulation above). Therefore, as gene expressions change over time, all requisite conditions must be satisfied throughout the network for it to attain a steady state. In this regard, the genome operates as a dynamic system of multiple interacting elements with feedback regulation, from which different self-stabilizing states, referred to as “attractor” states, are generated [74].

An important feature of an attractor state is that similar but less stable states in its neighborhood or “basin of attraction” tend to move—or are attracted—towards it. A corollary to this is that following a small displacement to another state within its basin, the system will return to its original attractor state. The movement towards established attractor states facilitates the adaptation of select gene expressions towards physiologically stable states, or cell phenotypes, over evolutionary time. Waddington introduced the term “canalization” to capture two essential requirements for development to proceed in an orderly coordinated fashion: first, a buffering potential ensures that organisms develop normally under a range of external conditions; second, the robustness of developmental decisions assures that choices are determined in an all-or-nothing manner rather than drift around [75]. Notwithstanding, stochastic fluctuations of gene regulators, like transcription factors and epigenetic regulators, or external stimuli, like growth factors, can influence gene expression programs and change the trajectory of cell development [76,77]. In effect, the cell is pushed to a position further away, and, if the force is vigorous enough, it can cross over the boundaries of its basin of attraction into the adjacent attractor. This genomic plasticity lays the foundation for differentiation by which a cell transitions from the pluripotent state through multiple progenitor states to the differentiated state. At the same time, the intrinsic robustness of the GRN provides a counterbalance that enables a cell to resist small changes in internal cues or external stimuli [78]. Under certain conditions, alterations of the gene expression programs are severe enough to be disruptive. The implications of this for cancer development are discussed in the next section.

## 6. From Normalcy to Malignancy: Role of Non-Coding RNAs

Metazoan development starts with the reprogramming of the zygotic genome into a transient totipotent state, which, in bilaterians, creates three germ layers (ectoderm, mesoderm and endoderm) that give rise to all cells in the organism. In humans, this comprises about 250 different cell types, totaling an estimated 4 × 10^13^ in the adult. A crucial property of epithelial and mesenchymal cells during embryogenesis is their ability to migrate to distant sites or form complex organs during morphogenesis [79]. This is directed by the Epithelial-to-Mesenchymal Transition (EMT) program, a cellular process that transiently converts epithelial into mesenchymal characteristics.

The events that unfold during transcription are crucial to developmental programs because they dictate the spatiotemporal expression of genes, which ultimately determines the cell type-specific proteome. While genes hold the blueprint for development and proteins are responsible for structure, ncRNAs provide an indispensable interface between the two to guide proper regulation of gene expression and cellular identity. Cell development begins at the level of the chromatin where epigenetic processes, such as DNA methylation and histone modification, control gene activation or repression [80,81]. Recruitment of RNA polymerase II (Pol II) initiates transcription, which occurs bidirectionally with sense mRNA and antisense lncRNA. Their expression from their own genes allows lncRNAs to regulate themselves [82]. More recent evidence suggests that transcription of lncRNA may be initiated by unidentified initiation factors and is *trans*-regulated by lncRNAs from other chromosomes [83].

In the nucleus, lncRNAs contribute to gene expression in different ways [29,30]. They modulate chromatin function and regulate the assembly and function of membrane-less nuclear bodies; they establish active and repressive domains through transcription repressor complexes; and they serve as scaffolds to tether enzyme complexes to gene promoters, which is followed by recruitment of transcription factors and transcription factor-DNA binding motif sequences within enhancers and promoters [84]. In addition to lncRNAs, various nuclear miRNAs participate in the regulation of gene transcription [85]. In the cytoplasm, miRNAs fine-tune protein synthesis through interactions with mRNAs [30].

**Figure 4:**
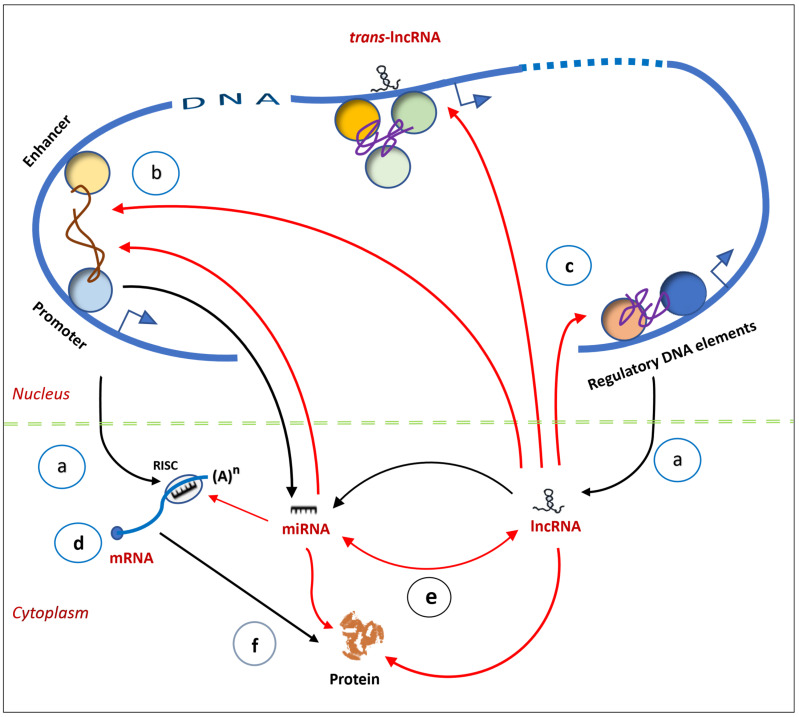
Schematic representation of ncRNA interactions with DNA, RNA and protein at the transcriptional and post-transcriptional levels. (a) Messenger RNAs (mRNAs) are transcribed from protein-coding genes while non-coding RNAs (ncRNAs) arise from multiple loci (including protein-coding genes). (b) ncRNAs play a critical role in organization of the genome and mediate promoter–enhancer interactions to coordinate gene expression. (c) Various ncRNAs coordinate gene expression, either locally (*cis*-acting) or at multiple gene loci (*trans*-acting). (d) Messenger RNA is subjected to regulation by miRNAs in the cytoplasm. (e) miRNAs and lncRNAs modulate each other through crosstalk. (f) miRNA and lncRNA interact with proteins to activate or repress them. (Biosynthetic pathways are indicated by black arrows, and ncRNA regulatory interactions are in red.) (miRNA—microRNA; lncRNA—long non-coding RNA; RISC—RNA-induced silencing complex.)

The capacity of ncRNAs to act locally as well as affect genomic segments separated by long linear distances on the same chromosomes or even on different chromosomes extends their regulatory reach across the genome [86,87]. Another important feature is the interplay among miRNAs, lncRNAs and circRNAs, which affects their function. For example, lncRNAs act as decoys by capturing miRNAs, thus preventing them from binding to their respective mRNAs [30,31,59]. In addition, ncRNAs interact with DNA and proteins to modulate epigenetic components, mediate intra-chromosomal interactions, and control gene transcription [88]. The result is an elaborate regulatory network established through ncRNA-ncRNA, ncRNA-DNA and ncRNA–protein connectivity (Figure 4). They form a parallel-processing system, whose signals are processed in many different pathways simultaneously, and they are non-linear and modifiable [89].

### 6.1. Consequences of Disruption of Non-Coding RNAs

Mutations of ncRNAs can lead to changes in gene-expression patterns and alterations of regulatory protein activities. Because the GRN is robust, minor degrees of rewiring of its components are tolerated, but the accumulation of a large set of genomic changes warps the state space and reshapes its contour. The corridor of accessible attractors is expanded as new developmental pathways are carved out. The outcome is that the susceptible cell can veer into other attractor states, among which might be previously unoccupied “cancer” attractors. This becomes an incipient cancer cell.

ncRNA functions can be disrupted in several ways. Single nucleotide variants (SNVs) in lncRNA alter its secondary configuration and interfere with lncRNA-miRNA interactions [90]. Also, SNVs in the 5′ UTR of *cis*-regulatory sequences affect translation, while those in the 3′ UTR disrupt RNA-RNA or RNA-protein interactions and, thus, interfere with post-transcriptional gene expression [91,92]. Further, in most cancers, ncRNAs directly or indirectly interact with oncogenic proteins, enforcing or dampening their action [93,94]. Chromosomal translocation, a frequent occurrence in cancer, can lead to aberrant fusion circRNA, by the back-splicing of complimentary repetitive intronic elements (Alu elements) [32]. Unlike normal circRNAs, which act as sponges, fusion circRNAs can abnormally stimulate cell proliferation [95]. In some cancers, adenosine-to-inosine (A-to-I) RNA editing in 3′ UTRs result in deletion of regulatory elements, like miRNAs, that they may contain; this loss can cause inappropriate oncogene activation [96]. Finally, chromosomal loss and duplication change the number of copies of ncRNAs.

### 6.2. Clinicopathological Considerations of the Cancer Attractor State

It is instructive to inquire how the concept of the cancer attractor reconciles clinical observations that are not fully explained. First, almost all cancers harbor a wide diversity of mutations despite having similar histological characteristics. For example, in small-cell lung cancer, which makes up about 15% of all lung cancers, several different oncogenic pathways are activated or silenced [97]. These include overexpression of the *MYC* family of oncogenes, dual inactivation of the tumor suppressor genes *TP53* and *RB1*, activation of the PI3K/Akt/mTOR pathway, and upregulation of the Notch pathway. Nonetheless, their histological appearance is similar. These observations can be explained by the convergence of different pathways toward a common cancer phenotype. Stated another way, the GRN steers the cell along trajectories within its state space into the basin of attraction of a particular attractor state.

Second, metastasis, a common occurrence in cancer patients, is an incompletely understood process [98]. To progress through the metastatic cascade, a cancer cell goes through a series of steps: local invasion and intravasation into the blood circulation, survival in the blood stream, and extravasation and growth in new organs [99]. Metastasis is ascribed to the known cancer genes and their associated signaling pathways that promote cell proliferation [100]. In addition to the proclivity for metastasis, a related issue is its timing. Although metastasis is generally believed to be a “late” event, early metastasis is frequent in the course of many cancers. In small-cell lung cancer, for example, about 70% of patients have overt metastatic disease at diagnosis, commonly in the lymph nodes, brain, liver and bones [97]. Similarly, in colorectal cancer, dissemination of cancer cells occurs early, even before the cancer is clinically detectable (<10 mm^3^) [101].

**Figure 5:**
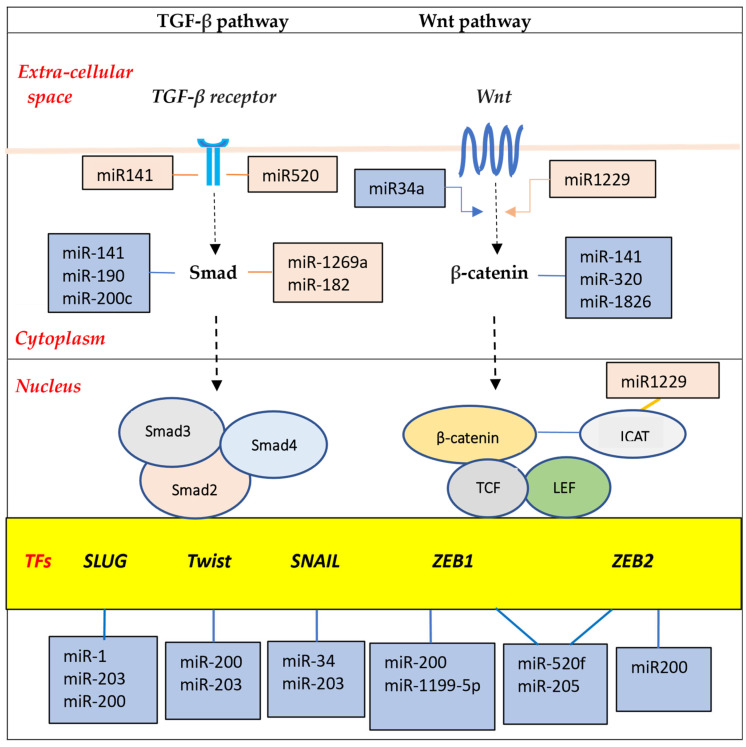
Schematic representation of TGF-β receptor and Wnt pathways, indicating the role of miRNAs in Epithelial-to-Mesenchymal Transition (EMT) regulation. Blue boxes indicate EMT inhibitory roles by miRNA, and pink boxes indicate induction of EMT (miR refers to the mature form of microRNA; TF—transcription factor).

It appears that at the core of the metastatic process is the Epithelial-to-Mesenchymal Transition (EMT) program [102,103]. This developmental program is essential for embryogenesis and tissue regeneration (e.g., wound healing), but its aberrant reactivation in cancer results in cells that are more mobile and invasive, and capable of overcoming the metastatic hurdles [104]. Several different signaling pathways, including TGFβ/Smad, WNT/β-catenin, Notch and tyrosine signaling pathways, can be activated in EMT programs [105,106,107]. These promote expression of EMT transcription factors, such as the zinc-finger E-box-binding homeobox factors ZEB1 and ZEB2, the zinc-finger factors SNAI1 (SNAIL) and SNAI2 (SLUG), and the basic helix–loop–helix factors TWIST1 and TWIST2 (Figure 5).

Importantly, various miRNAs interact in complex ways with these transcription factors to drive or buffer EMT signal pathways. For example, miR-200 and miR-205 cooperate to suppress ZEB1/2 expression; on the other hand, ZEB1/2 directly suppress the transcription of the miR-200 [108]. Similarly, a mutually inhibitory loop is present between Zeb1 and miR-1199-5p. These double-negative feedback loops serve as epithelial/mesenchymal switches to enhance the versality required by cells for EMT. Additionally, a number of lncRNAs regulate EMT through various mechanisms, such as functioning as competitive endogenous RNA (ceRNA) to sequester miRNA, or sponging of miRNAs. Further, lncRNAs interact with transcription factors to direct the polycomb-repressive complex 2 (PRC2) to various epithelial gene targets [94,109,110,111].

The epithelial and mesenchymal cells correspond to distinct attractor states under the tight control of ncRNAs [112,113,114]. However, instead of transitioning between the epithelial and mesenchymal states, cancer cells undergo a partial or transient EMT, displaying a phenotype with a mixture of epithelial and mesenchymal traits [115]. The hybrid E/M cells have a higher potential for metastasis compared with cells at either end of the EMT spectrum. Additionally, they display increased cancer stem cell activity or pluripotency [103]. In summary, the hijacking of the EMT program by the cancer cell generates a hybrid E/M attractor state, which endows the cell with highly malignant traits, including a mechanism for metastasis at early stages.

## 7. Discussion

The most conspicuous anomaly in cancer cells is multiple mutations of oncogenes and tumor suppressor genes, which often result in constitutive activation of signaling protein cascades that promote cell proliferation. Although not all mutations are necessarily compatible with cell survival, those that are tolerated could confer a selective growth advantage over its normal counterpart [116]. As posited by the somatic mutation theory, this yields an expanding clone of cells that acquire progressive malignant traits. But the observed gene mutations are inconsistent among cancers. Beyond cancer, many of these mutations are also associated with benign conditions and are prevalent in healthy tissues. The discrepancy among the putative cancer genes and their functional diverseness suggest that other mechanisms are at play during the development of a cancer [1,2,3].

Alongside the overt chromosomal abnormalities observed in cancer cells, there is a large, hidden layer of ncRNAs. They operate at several different cellular levels, from transcriptional control of gene expression through post-transcriptional modulation of mRNA stability to post-translational functioning of proteins. Through their crosstalk with other RNAs as well as their interplay with DNA and proteins, they are integral components of regulatory mechanisms that have previously been attributed to proteins. This discovery has led to a new way of thinking about how the genome is organized, and represents a conceptual shift about how a cancer might develop [117,118].

An appreciation of the central role of the genome regulatory system in tumorigenesis starts with the recognition that cells are defined by their gene-expression patterns as part of a dynamic network of interacting elements. The metazoan genome contains a huge number of possible gene configurations, which are either persistent and stable, or evanescent and unstable. From any starting point, the system spontaneously evolves and eventually settles down into stable attractor states, corresponding to distinct cell types [69,74,119]. Over time, cell development has become canalized in such a way that cells are guided by evolutionary maps down narrow valleys in the epigenetic landscape to those attractor states that serve useful physiological functions [75]. Nonetheless, within the GRN state space, other potential attractor states exist, but are not normally occupied, since the less functional attractors are bypassed. (It is germane to note here that the term “epigenetic landscape” is used as a metaphor, as introduced by Waddington, to refer to a system-level stable state of genetic interactions rather than the more common concept of “epigenetic trait” as a heritable phenotype resulting from changes in a chromosome without alterations in the DNA sequence [120].)

An important property of the attractor state is its robustness to tolerate minor changes. However, a collection of multiple disruptions of ncRNAs could effectively rewire the GRN and push the cell towards a different attractor state or cell phenotype. In consonance with the notion of the cell as an attractor state, the cancer cell, whose behaviors are conditioned by the particular pattern of the connections and interactions within its regulatory network, also represents an attractor state [73,89]. Eukaryotes are enriched in ncRNAs, which are involved in the regulation of genomic output at different levels. Dysregulation of ncRNA interactions and dynamics can warp the topography of the epigenetic landscape and cause the cell to veer towards a cancer attractor.

This view offers a different perspective of the role of somatic mutations of oncogenes and tumor suppressor genes in cancer pathogenesis. Alterations of these genes contribute to the proliferation of cancer cells through their action on the cell cycle apparatus and their links to associated signal transduction pathways. Moreover, different ncRNA circuitries intersect with oncogenic pathways, which can be indirectly activated or repressed. However, the noteworthy consideration is that the presence of mutated oncogenic driver genes appears insufficient to cause cancer. Rather, this transition is transacted at the level of the incipient cancer cell with its immanent neoplastic program scripted by regulatory ncRNAs.

### Implications for Systemic Cancer Therapy

The centrality of the ncRNA network in tumorigenesis has some implications regarding our approach to systemic cancer therapy. Cytotoxic chemotherapy was introduced in the 1940s on the assumption that the mitotic cycle is perturbed in cancer cells, and, in the late 1990s, the discovery of disrupted oncogenic signaling pathways guided the design of molecularly targeted therapy [121,122]. Both of these therapies have improved the cure rates of several types of cancer when given in the post-operative adjuvant setting after resection of all macroscopic disease, and also benefited patients with metastatic disease. Nonetheless, patients with advanced or metastatic cancers, especially those originating in epithelial tissues like breast, lung, prostate or colorectum, have modest extension of their overall survival times, but cures are still usually unattainable [123].

The emerging information about the role of ncRNAs in the cell’s genomic regulatory system is beginning to influence the next generation of cancer therapy [124]. The targeting of disrupted ncRNAs by nucleic acid-based therapy could herald a different strategy to the design of novel anti-cancer drugs [125]. But new opportunities also bring new challenges. If multiple alterations of the ncRNA regulatory network are involved in the cancer process, we can assume that restoring order would require several points of intervention. To accomplish this, a detailed understanding of ncRNA dynamics and how specific components impact overall network functioning is necessary [126,127]. Further, while data on ncRNA localization and connectivity might capture the activities of ncRNAs in their native cellular environments, their wide-spread interactions with other RNA transcripts, DNA and RNA-binding proteins can be affected by various factors, such as accessibility to binding sites, ncRNA secondary and tertiary structural conformations, and post-transcriptional modifications. In this regard, the use of deep-learning approaches to infer biologically meaningful ncRNA structure and predict their interactions should accelerate the development of nucleic acid-based therapy [28,128].

In conclusion, the evolutionary progress from simple prokaryotes to the complex eukaryotes is orchestrated by an elaborate system of regulatory ncRNAs, which are conserved and distributed across all three domains of life. They are the architects of cellular complexity in multicellular organisms, but when they misfire, they become the agents of malignancy. The new understanding of the organization and regulation of the genome affords us an opportunity to update our views about the early stages of cancer development to better align with research findings and clinical observations.

## Data Availability

No new data were created in this review.

## Abbreviations

The following abbreviations are used in this manuscript:

3′ UTR	3′ untranslated region
5′ UTR	5′ untranslated region
ceRNA	competitive endogenous RNA
circRNA	circular RNA
EMT	Epithelial-to-Mesenchymal Transition
GRN	genome-wide regulatory network
lncRNA	long non-coding RNA
miR	mature form of microRNA
miRNA	microRNA
mRNA	messenger RNA
ncRNA	non-coding RNA
nt	nucleotide
PRC2	polycomb-repressive complex 2
RBS	ribosomal binding site
RISC	RNA-induced silencing complex
rRNA	ribosomal RNA
snoRNA	small nucleolar RNA
SNV	single nucleotide variant
sRNA	small RNA
tRNA	transfer RNA
